# Palliative Percutaneous Gastrostomy Decompression Methods for Small-Bowel Obstruction in Advanced Gastrointestinal Cancer

**DOI:** 10.3390/cancers17081287

**Published:** 2025-04-10

**Authors:** Ahmed Alwali, Clemens Schafmayer

**Affiliations:** Department of General, Visceral, Vascular, Thoracic, and Transplantation Surgery, Rostock University Medical Center, 18057 Rostock, Germany

**Keywords:** malignant bowel obstruction (MBO), palliative decompression, percutaneous endoscopic gastrostomy (PEG), transhepatic, transesophageal, venting gastrostomy

## Abstract

Background: Malignant bowel obstruction (MBO) is a common and distressing complication in advanced gastrointestinal cancers, significantly impacting patients’ quality of life. When conservative management fails, palliative decompression is essential to relieve symptoms such as nausea, vomiting, and abdominal distension. Venting gastrostomy is the most established method; however, anatomical challenges may necessitate alternative percutaneous approaches. Objective: This narrative review aims to provide a comprehensive overview of percutaneous gastrostomy techniques for palliative gastrointestinal decompression, including percutaneous endoscopic gastrostomy (PEG), interdisciplinary imaging-guided percutaneous or transhepatic gastrostomy, and percutaneous transesophageal gastrostomy (PTEG). Methods: A literature review was conducted to evaluate the indications, techniques, efficacy, and complications associated with these procedures. The role of a multidisciplinary approach, incorporating radiologic, endoscopic, and palliative care expertise, was also explored. Results: PEG remains the gold standard for venting gastrostomy, achieving symptom relief in up to 92% of cases, with a low complication rate. However, interdisciplinary imaging-guided percutaneous or transhepatic gastrostomy offers a viable alternative for patients with surgically altered anatomy or difficult percutaneous access. PTEG, a newer technique, has demonstrated high technical success and symptom improvement, particularly in patients with extensive peritoneal carcinomatosis or massive ascites, where transabdominal approaches are not feasible. Conclusions: Palliative percutaneous decompression provides effective symptom relief in advanced gastrointestinal cancer. The choice of technique should be individualized based on patient anatomy, clinical condition, and resource availability. A multidisciplinary approach remains crucial in tailoring decompression strategies to improve the quality of life in end-stage malignancies.

## Simple Summary

Patients with advanced gastrointestinal cancers often develop malignant bowel obstruction, which causes severe discomfort, including nausea, vomiting, and abdominal pain. When conservative therapy fails and a surgery is no longer an option, doctors use alternative techniques to relieve symptoms and improve quality of life. One of the most common methods is inserting a tube through abdominal wall into the stomach (gastrostomy) to drain fluid and gas, reducing pressure and discomfort. However, traditional approaches may not be possible for some patients due to changes in anatomy from previous surgeries or cancer spread. This review discusses different gastrostomy techniques that can be used when standard procedures are not suitable. By evaluating these options, we aim to help healthcare professionals choose the best technique for each patient. Expanding access to these procedures may improve symptom control and overall comfort for individuals with advanced cancer.

## 1. Introduction

Advanced gastrointestinal cancers frequently result in bowel obstruction, leading to debilitating symptoms such as nausea, vomiting, and abdominal distension. Malignant bowel obstruction (MBO) occurs in approximately 3–15% of end-stage cancer patients [1]. Approximately 10–28% of gastrointestinal cancer patients develop an MBO, with around 15% of terminal-stage patients on a palliative care unit diagnosed with it [2,3]. The prognosis is poor, with median survival post-diagnosis ranging from weeks to a few months [3]. Pathogenetically, a distinction is made between mechanical obstruction and functional obstruction, also known as paralytic obstruction. Proper diagnosis is crucial for therapy, as functional causes are more manageable with conservative treatment compared to mechanical obstructions. Tumor growth can lead to an ileus through both direct and indirect mechanisms. A locally advanced tumor causes a direct intraluminal obstruction of the intestine, while extraluminal tumor masses can exert pressure on the intestinal lumen from the outside. Indirectly, tumor infiltration of the intestinal musculature can impair bowel motility, as can tumor infiltration into the mesentery, the celiac plexus, or other nerve structures. Small-bowel obstructions are most often caused by peritoneal carcinomatosis [4].

Inflammatory edema formation, constipation, reduced intestinal secretion, changes in gut flora, and medication side effects—particularly from opioids, tricyclic antidepressants, anticholinergics, or neuroleptics—can cause a mechanically insignificant obstruction to acquire functional relevance [5]. Furthermore, even in advanced malignant underlying diseases, there is a significant rate (3–48%) of non-malignant causes of bowel obstruction, such as adhesions, radiation-induced strictures, internal herniations, and electrolyte imbalances like hypercalcemia or hypokalemia. These non-malignant causes are clinically indistinguishable from malignant ones. The dilation of the intestinal wall associated with the passage disorder leads to increased intestinal contractions, which can be experienced as colic. The enlargement of the epithelial surface results in increased secretion, intensifying symptoms such as nausea and vomiting. The stretching of the epithelial surface causes inflammatory epithelial damage, leading to intestinal wall edema, hyperemia, and the production of prostaglandins, vasoactive intestinal peptide (VIP), and nociceptive mediators, which explain the persistent pain component.

The symptoms of MBO in tumor patients usually develop gradually over days or weeks, often initially occurring intermittently. As the condition progresses, episodes become more frequent and intense, potentially leading to complete obstruction [6].

The treatment of MBO represents one of the greatest challenges in palliative medicine and is usually managed in an inpatient setting. Defining the treatment goal is crucial for medical success and for patient and family satisfaction. The primary objective is symptom control to improve quality of life. For a long time, survival time was the primary endpoint in studies. However, it is now widely accepted that survival duration is of lesser importance as an outcome measure [7,8]. Instead, parameters such as relief from nausea, vomiting, and pain; the ability to consume solid or oral nutrition; the restoration of bowel function; and the possibility of being discharged home are more relevant in a palliative setting [9].

It is particularly important to recognize that for many patients and their loved ones, MBO represents their first confrontation with impending death. The strength of a multidisciplinary palliative care team becomes especially evident in such situations. Studies have shown that feelings of social isolation or recurrent severe depressive episodes are associated with increased mortality, a burden that palliative care teams can help mitigate [10]. MBO is rarely a medical emergency. Nausea, vomiting, and pain are often significantly reduced by bowel rest, pharmacologic therapy (antiemetics, anticholinergics, octreotide, corticosteroids), and nasogastric tube decompression. In some cases, the placement of a nasogastric tube along with fluid resuscitation can resolve the intestinal obstruction, with a retrospective study reporting an average resolution time of approximately 9 days [11]. However, nasogastric tubes offer only short-term relief, and their prolonged use is associated with significant discomfort and complications such as nasal erosion, bleeding, and aspiration [12].

Surgical intervention for malignant intestinal obstruction in advanced cancer stages is rarely effective. Multi-segmental compression is common, making rapid re-compression likely even if a stenosis is surgically treated [13,14,15]. Additionally, poor patient performance status and widespread disease contribute to high morbidity and mortality associated with surgery. The operative mortality rate ranges from 5% to 32%, morbidity is 42%, and the rate of re-stenosis varies between 10% and 50% [3,15,16]. In a recent randomized study, the in-patient complication rate was higher in the surgery arm compared to the non-surgical arm (23% vs. 12%), though the length of the initial hospital stay remained similar across both groups [17]. Given these significant risks, the burden of surgical complications extends beyond immediate postoperative concerns. Surgical morbidity may prolong hospitalization, further compromising quality of life. More critically, surgical intervention may accelerate death, representing the most adverse outcome. As a result, non-surgical palliative measures are generally preferred for most patients with advanced intra-abdominal cancer. Consensus guidelines recommend managing patients with MBO unsuitable for surgery with a venting gastrostomy if they do not respond to medical treatment [18].

Palliative gastric decompression focuses on draining accumulated gastrointestinal contents proximal to an obstruction, thereby alleviating symptoms and reducing complications such as aspiration or bowel perforation. This approach often allows patients in end-of-life care to resume small amounts of oral intake for comfort. The indications for a percutaneous gastric decompression in malignancy are obstructions proximal to the mid–small-bowel (e.g., gastric outlet, duodenum, proximal jejunum) or multifocal bowel obstructions where upstream decompression is needed for symptom relief.

Currently, three percutaneous approaches are reported:

- Percutaneous endoscopic gastrostomy (PEG)
- Interdisciplinary imaging-guided percutaneous/transhepatic gastrostomy
- Percutaneous transesophageal gastrostomy (PTEG)

This narrative review aims to compile, assess, and synthesize information from various sources to provide a comprehensive overview of percutaneous gastrostomy techniques for palliative gastrointestinal decompression in end-stage malignant small-bowel obstruction.

## 2. Methods

This review aims to present the reported palliative gastrostomy decompression methods for MBO in advanced gastrointestinal cancer mentioned in the literature, with a particular focus on non-operative techniques utilizing percutaneous access. While not all individual studies were included, we considered systematic reviews on palliative decompression, as well as reports where percutaneous gastrostomy was used for nutritional support in complex anatomical scenarios, given its potential applicability for decompression. This is a traditional narrative review rather than a systematic review, designed to explore and discuss various gastrostomy techniques for palliative decompression. We conducted an extensive literature search from 1990 to the present, incorporating the most recent systematic reviews when available. The search strategy involved the use of key terms, including malignant bowel obstruction (MBO), palliative decompression, percutaneous endoscopic gastrostomy (PEG), and transhepatic, transesophageal, and venting gastrostomy in databases such as Medline, Embase, and PubMed. Special attention was given to studies reporting gastrostomy techniques used in cases of altered anatomy or challenging clinical situations. In instances where only a limited number of articles described a specific method for gastrostomy in complex anatomical scenarios, these were included to ensure comprehensive coverage of available techniques.

## 3. Percutaneous Endoscopic Gastrostomy (PEG)

Percutaneous endoscopic gastrostomy tubes were initially developed as a method for enteral nutrition, introduced by Ponsky and Gauderer in 1980 as a less invasive alternative to surgical gastrostomy [19]. Their application later expanded, with Malone et al. describing percutaneous radiologic gastrostomy (PRG) for gastric decompression in 1986 [20]. The technique was soon adapted, and in 1987, Stellato and Gauderer documented the first use of PEG specifically for decompression, establishing its role in managing gastric distension and related conditions [21]. PEG is now one of the most commonly used palliative treatment procedures. Endoscopic gastrostomy achieves symptom control in 84% to 92% of patients and can facilitate the resumption of comfort oral intake [22,23,24,25,26]. A large prospective study by Zucchi et al. further supports the effectiveness of decompressive PEG in patients with advanced malignancy and small-bowel obstruction. Among 158 patients, PEG was successfully placed in 89.8%, with 77.4% experiencing relief from nausea and vomiting within 48 h. Quality of life improved in 64% of patients evaluated using the Symptom Distress Scale, and over 80% were discharged after PEG placement [27]. A recent systematic review evaluating the effectiveness of venting gastrostomy in 1194 patients with malignant bowel obstruction reported a 92% symptom relief rate, with 84% of patients able to tolerate oral intake, an important contributor to quality of life, by intermittently clamping the tube, thereby preserving the psychosocial pleasure of eating and allowing patients to be involved in family meals and not socially isolated [23]. The insertion failure rate was 9%, and the median survival in this cohort ranged from 14 to 74 days, aligning with findings from previous studies. PEG also allows for the discontinuation of nasogastric tubes, which are often uncomfortable and pose restraints on patients; this enables patients to mobilize and potentially be discharged from hospital to home or hospice care with the gastrostomy tube in place for ongoing management.

Technique: Venting gastrostomy involves transabdominal placement of a tube into the stomach for the purpose of continuous drainage of luminal contents and gas. The tube is most often placed percutaneously under endoscopic guidance. In brief, under conscious sedation, an endoscope is introduced into the stomach to insufflate it and transilluminate the abdominal wall. A suitable site on the epigastrium is identified (often in the upper-left quadrant) where the illuminated stomach is apposed against the peritoneum. The “pull” (Ponsky–Gauderer) technique or “push” (Russell) technique is then employed [19,28]. The tube is secured internally by a retention bumper or balloon and externally by a fixation device. In the pull technique, a small cutaneous incision is made down to the fascia. A catheter-over-needle is then inserted percutaneously into the stomach, and a snare or a biopsy forceps is advanced through the endoscope. In the next step, the needle is removed, and a silk suture loop (or “string”) is threaded through the catheter into the stomach. The endoscopic snare captures the string and pulls it out through the mouth via the endoscope. The wire loop of the string is then secured to the wire loop of the PEG tube. Using the string as a guide, the PEG tube is drawn back through the mouth, into the stomach, and finally out through the abdominal wall. Some protocols include gastropexy (suturing the stomach to the abdominal wall with suture Freka Pexact II, Fresenius Kabi GmbH, Germany, or SAF-T-PEXY T-fasteners, Avanos, Alpharetta, GA, USA) to secure apposition and reduce leak risk [29].

The push technique is primarily used for fluoroscopic placement by radiologists but can also be performed by an endoscopist, especially in case of neck tumors or advanced ascites. The initial steps are similar, involving the placement of a trocar into the stomach under endoscopic visualization, followed by the insertion of a guidewire into the stomach. The guidewire is then secured using an endoscopic snare. Next, two to four T-fasteners or sutures, using a Freka Pexact II device, are deployed around the trocar through a cannula to achieve gastropexy. Finally, the tract through which the guidewire passes is gradually dilated, and a peel-away sheath is introduced over the wire [30]. While technique application varies by institution, the pull technique may have lower adverse event rates in non-oropharyngeal cancer patients, particularly in palliative decompression [31]. Major complications—such as intraperitoneal hemorrhage, visceral perforation, or peritonitis—are reported in a small percentage of cases (<5%) [22]. Patient-related factors often drive outcomes more than technical failures. Advanced cancer patients are inherently vulnerable: one analysis found that a high ASA score and advanced tumor stage were independent predictors of worse overall outcomes after PEG in cancer [22]. Aspiration related to the gastrostomy tube procedure occurs in approximately 0.3% to 1% of cases. Minor infections are relatively common, with an incidence ranging from 5.4% to 30%. Acute bleeding is a rare complication, occurring in about 1% of cases, with fewer than 0.5% requiring blood transfusion or laparotomy for bleeding control [32]. Inadvertent perforation of the intestines is a rare but potentially fatal complication. Excessive gastric and small-bowel insufflation may result in bowel transposition and gastric rotation. Proper transillumination and positioning in reverse Trendelenburg may help mitigate this risk. Tube dislocation is another concern, as the gastrostomy tract typically matures within seven to ten days but may take longer in patients with malnutrition, ascites, or those undergoing steroid treatment. If the tube becomes dislodged during this period, it can lead to free perforation. The use of gastropexy devices at the time of tube placement may help secure the tube and reduce the risk of complications [33,34].

In summary, percutaneous endoscopic gastrostomy is a safe palliative intervention with a well-defined complication profile, primarily minor issues, and a low risk of serious events, making it a favorable option for malignant GI obstruction relief.

## 4. Interdisciplinary Imaging-Guided Percutaneous/Transhepatic Decompression

Despite the advantages of PEG, certain patients present significant challenges to conventional endoscopic and radiologic approaches. In prior series, patients without a safe percutaneous access route to the stomach were often excluded from these procedures. In such complex cases, computed tomography (CT) has proven valuable in identifying an optimal pathway for safe and effective gastrostomy tube placement, much like its role in guiding percutaneous abscess drainage [35,36,37,38,39]. By providing detailed anatomical visualization, CT guidance offers an alternative approach when conventional methods are not feasible. A recent systematic review reported technical success rates of 82–100% for decompressive gastrostomies performed under CT guidance. While symptom relief was not consistently assessed in the CT-guided subgroup, decompressive gastrostomies overall achieved symptom control in 77.4–92% of cases, with quality-of-life improvements in up to 64% of patients. CT-guided techniques were primarily used as a secondary option in patients where endoscopic placement had failed [37]. Additionally, incorporating endoscopic guidance within the CT scan room provides further assistance in facilitating accurate CT-guided puncture. This approach represents a reverse scenario of the conventional workflow, where CT typically guides surgical interventions. The integration of endoscopic expertise in the radiology suite underscores the critical role of interdisciplinary collaboration between endoscopists and radiologists. By combining real-time endoscopic visualization with precise CT imaging, this cooperative approach enhances procedural success and expands treatment options for patients with challenging anatomical conditions [40].

CT-guided percutaneous transhepatic decompression is described only in a few cases as a variation of percutaneous gastrostomy in altered anatomy, especially when the stomach remnant adheres to left lobe of the liver, likely due to concerns about potential bleeding complications and abscess formation [41,42]. Some authors suggest inadvertent hepatic injury is more frequent than reported but is usually well tolerated [43,44,45,46].

Transhepatic puncture is a known procedure in interventional radiology, frequently performed for hepatobiliary interventions and cholecystostomy in non-operable patients. This technique involves accessing the stomach by passing through the liver parenchyma, typically the left lobe. Kanazawa et al. first documented this approach in 1995, describing its use in four patients with partial gastrectomy who were unable to tolerate oral feeding [41]. Using CT guidance, the gastric remnant was punctured with a 22-gauge percutaneous transhepatic cholangiography needle via the left lobe of the liver. A guidewire was then introduced, followed by sequential tract dilation to accommodate 8F or 9F catheters, which were fluoroscopically positioned using Cope loop catheters. The procedures were completed without apparent complications, and catheter feeding was successfully maintained for two to seven months without major issues. Moriwaki et al. reported a successful transhepatic duodenostomy for enteral nutrition in a post-gastrectomy patient, where the remnant stomach was positioned behind the lateral liver segment, making standard PEG challenging [42]. Using a CT-guided transhepatic pull method with real-time ultrasound and endoscopic assistance, they carefully avoided intrahepatic vascular and biliary injury. After confirming needle placement via CT and endoscopy, a guidewire was introduced and a PEG tube was placed.

Interestingly, there have been reports of accidental transhepatic PEG tube placements in which the tube traversed the left lobe of the liver without causing significant immediate or long-term complications. In some cases, the PEG remained in place for several months and was eventually removed without issues, despite its unusual positioning [43,44,45,46]. In summary, interdisciplinary imaging-guided percutaneous/transhepatic decompression requires close collaboration between an interventional radiologist, an endoscopist, and a gastrointestinal surgeon. This approach appears to achieve the same therapeutic success as standard venting gastrostomy by effectively decompressing the stomach and upper intestines to relieve symptoms. Its value lies in making this decompression possible in patients who would otherwise have no good options. Given its technical success and symptom relief in small cohorts, the transhepatic approach is a viable option when performed by experienced clinicians.

## 5. Percutaneous Transesophageal Gastrostomy (PTEG)

**Figure 1:**
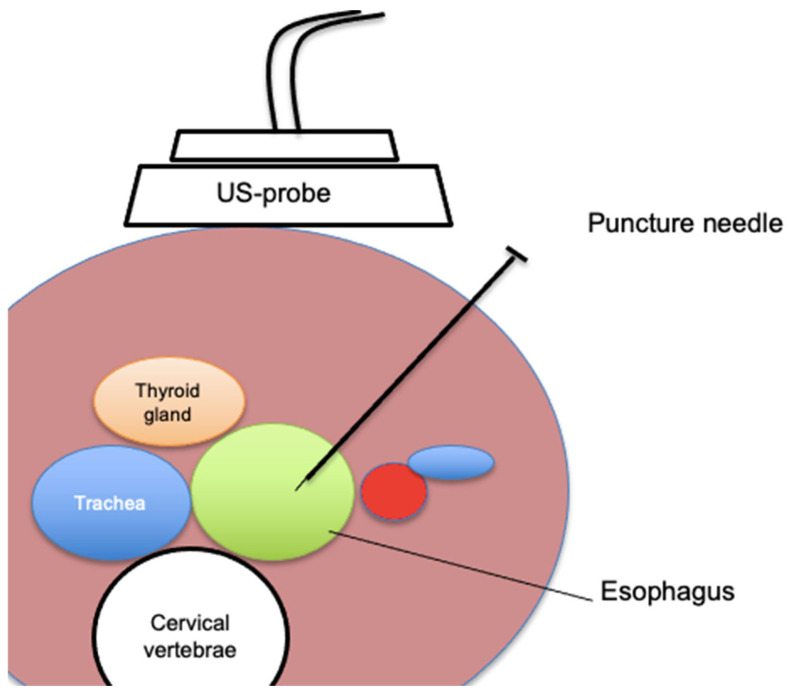
Schematic illustration of an ultrasound-guided puncture of the cervical esophagus.

Percutaneous transesophageal gastrostomy is a new intervention for palliative care patients for gastrointestinal decompression that was developed to circumvent the challenges of transabdominal gastrostomy in certain patients [47]. For clarity, the term “transesophageal gastrostomy” can be a bit confusing—the tube does terminate in the stomach, but enters via the esophagus rather than the skin of the abdomen. Some literature also calls the procedure percutaneous transesophageal gastro-tubing. Unlike a traditional gastrostomy that accesses the stomach through the abdominal wall, PTEG creates an esophagostomy in the cervical esophagus and places a tube through that route in the upper stomach. This technique was first described in the 1990s in Japan and has since seen slow adoption elsewhere [47,48,49,50,51]. Typically, the procedure is performed in an interventional radiology suite or endoscopy unit under fluoroscopic and ultrasound guidance. A specially designed rupture-free balloon catheter is inserted into the esophagus, usually via the nose or mouth, and positioned in the upper esophagus just below the cricothyroid junction (around the level of the sternal notch). This balloon is then inflated to distend the esophagus. Using ultrasound on the left side of the neck, the operator identifies the inflated balloon between the trachea and the carotid artery—the esophagus at this level lies slightly left of midline, providing a window for access. Through a small left neck incision, a needle is carefully advanced under ultrasound guidance into the esophagus, puncturing the balloon. A guidewire is then threaded through the needle into the esophagus and down into the stomach. Over this wire, the tract is sequentially dilated. Ultimately, a gastrostomy-type tube with an internal retention loop is placed through the neck and advanced such that the loop opens within the stomach and the tube resides with its side holes in the stomach. The external portion is secured at the neck with sutures to prevent displacement (Figure 1).

A recent multicenter case series of 24 patients undergoing PTEG for enteral feeding or palliative venting reported a 100% technical success rate. All patients had contraindications to normal gastrostomy. Minor postprocedural complications included local cellulitis, significant tube clogging, and dislodgement. One major complication occurred—a significant bleed at the catheter entry site—which was successfully managed with a carotid covered stent [48]. Similarly, Selby et al. documented successful PTEG insertions in all 10 cancer patients with MBO refractory to medical therapy, where standard venting gastrostomy was not an option. No acute postprocedural complications were observed. Because the PTEG tube exits from the neck and typically runs upright, passive drainage by gravity alone is often insufficient, necessitating continuous suction to maintain decompression. Patients did not report discomfort regarding the appearance of the PTEG. From a quality-of-life perspective, most emphasized the relief of having the nasogastric tube removed and the ability to drink fluids without experiencing nausea or vomiting [49]. The safety profile of transesophageal gastrostomy is generally favorable, but the procedure is not without risks. Having a tube exiting in the neck is unusual and can cause mild throat discomfort or require adjustments in head positioning. Toh Yoon et al. conducted an observational study evaluating PTEG as an alternative long-term feeding option when gastrostomy was unsuitable. Among 15 patients, complications included tube dislodgement (n = 3), tracheoesophageal fistula (n = 1), inferior thyroid artery injury (n = 1), and thyroid gland mispuncture (n = 1), but no procedure-related or 30-day mortality was observed [50].

Udomsawaengsup et al. reviewed the use of PTEG as a nonsurgical alternative for enteral access in patients with a hostile abdomen, altered gastric anatomy, massive ascites, or carcinomatosis. In their study of 17 terminally ill patients, primarily with metastatic cancer, PTEG placement was successful in 94% of cases. There were no major complications, while minor complications occurred in 17.6%, including two esophageal leaks and one catheter dislodgement. Despite a high one-month mortality rate (41.2%) due to underlying conditions, all patients were discharged with functional enteral access and gastrointestinal decompression [51]. A scoping review by Zhu et al. analyzed 14 studies involving 340 patients and confirmed that PTEG is a safe and effective alternative when standard gastrostomy is not feasible due to factors such as ascites or altered anatomy. The review reported a technical success rate of 98.8%, with minor complications in 19.1% and major complications in only 2.1%, including bleeding and aspiration pneumonia. Notably, nearly all patients experienced symptom relief, and the one randomized controlled trial included in the review demonstrated a significantly better quality of life with PTEG compared to with nasogastric tubes [52]. One limitation of PTEG is the requirement for specialized equipment and training, which may restrict its availability. Not all hospitals have PTEG kits or staff familiar with the technique. Additionally, managing the PTEG output typically involves continuous suction, as noted, which can tie the patient to a suction device—this is a logistical consideration for home or hospice care (portable suction machines can address this, but it is another layer of care). Patients and caregivers need education on caring for a neck stoma and handling the tube, which is different from the more familiar abdominal gastrostomy. There can be cosmetic or psychosocial implications, too: a tube in the neck might bother some patients, though many in the palliative setting are unperturbed if it relieves their agony from obstruction (Table 1).

In summary, although PTEG presents specific risks, particularly those associated with cervical access, its overall complication rate remains low, making it a viable alternative for managing complex MBO cases. Optimal patient selection, skilled operator technique, and diligent post-procedure care—especially maintaining continuous drainage—are essential for reducing complications and enhancing the palliative benefits of transesophageal gastrostomy.

## 6. Conclusions

Palliative decompression of the gastrointestinal tract is a key component of symptom management in advanced GI cancers with obstruction when conservative measures fail. For patients who are no longer candidates for curative or definitive surgical intervention, venting gastrostomy provides effective symptom relief and enhances quality of life in their terminal stages. As the most established approach, venting gastrostomy is simple, highly effective in reducing nausea and vomiting, and carries a low risk of serious complications. It should be considered for appropriate patients with malignant obstruction, as it significantly alleviates symptom burden and allows limited oral intake, which holds important social and psychological benefits. For those with anatomically challenging conditions such as surgically altered anatomy, interdisciplinary imaging-guided percutaneous or transhepatic venting gastrostomy provides a safe solution when performed carefully. This variation expands the utility of venting gastrostomies, enabling more patients to benefit from venting. Although data are limited, initial outcomes show that it achieves symptom relief comparable to standard PEG. Percutaneous transesophageal gastrostomy is a valuable recent addition for patients who truly cannot undergo transabdominal tube placement. It bypasses the peritoneum entirely, offering effective decompression in scenarios of dense carcinomatosis or massive ascites that would preclude other methods. PTEG has demonstrated high technical success and symptom improvement rates, reinforcing that it should be considered a go-to option in specialized centers for otherwise untreatable obstructions. Each modality has particular advantages and drawbacks, and the choice must be individualized based on patient anatomy, clinical condition, and resource availability. From a clinical practice standpoint, a multidisciplinary approach is essential—input from palliative care, oncology, gastroenterology, and interventional radiology will ensure that the chosen decompression method aligns with the patient’s goals and the medical realities. In conclusion, palliative percutaneous decompression offers significant symptom relief in advanced gastrointestinal cancer and remains a critical intervention.

## Figures and Tables

**Table 1:** Comparative table of decompression techniques.

	PEG	PTEG	CT-/Imaging-Guided/Gastrostomy
Patient Comfort	Generally well tolerated	Neck tube may cause discomfort	Similar to PEG if successful; abdominal tube placement
Quality-of-Life Metrics	Improves nausea, vomiting; allows oral intake	Fewer data on QoL	Similar to PEG
Overall Complication Rate	Minor: 5–30%; major: <5%	Minor: ~18%; major: ~2%	Minor: 5–30%; major: ~5–10% (varies by anatomy)
Training and Equipment Required	Endoscopy, basic sedation	Personal experience, fluoroscopy, ultrasound guidance	Advanced CT suite with interventional experience
Patient and Caregiver Education	Relatively straightforward	Tube in neck requires explanation, suction devices required	Similar to PEG
Technical Complexity	Moderate; well-established technique	High; cervical access, uncommon technique	High; requires personal experience
Patient Selection	Favorable anatomy	Difficult abdominal access, hostile peritoneum	Prior gastrectomy, failed PEG
Limitations	Not feasible in altered anatomy or absence of transillumination	Hardly any experience	Availability limited to tertiary centers with trained teams

PEG: percutaneous endoscopic gastrostomy, PTEG: percutaneous transesophageal gastrostomy.

## Data Availability

Not applicable.

## Abbreviations

The following abbreviations are used in this manuscript:

MBO	Malignant bowel obstruction
PEG	Percutaneous endoscopic gastrostomy
PTEG	Percutaneous transesophageal gastrostomy
CT	Computed tomography
ASA	American Society of Anesthesiologists
